# The Effect of the Post 2001 Reforms on FMD Risks of the International Live Animal Trade

**DOI:** 10.1007/s10393-018-1315-8

**Published:** 2018-02-27

**Authors:** David W. Shanafelt, C. Perrings

**Affiliations:** 1 Centre for Biodiversity, Theory and Modelling, Theoretical and Experimental Ecology Station, UMR 5321, CNRS and Paul Sabatier University, 2 Route de CNRS, 09200 Moulis, France; 2 School of Life Sciences, Arizona State University, Tempe, AZ USA

**Keywords:** Foot and mouth disease, International trade, Risk

## Abstract

The 2001 UK foot and mouth disease (FMD) epidemic marked a change in global FMD management, focusing less on trade isolation than on biosecurity within countries where FMD is endemic. Post 2001 policy calls for the isolation of disease-free zones in FMD-endemic countries, while increasing the opportunities for trade. The impact of the change on disease risk has yet to be tested. In this paper, we estimate an empirical model of disease risk that tests for the impact of trade volumes before and after 2001, controlling for biosecurity measures. In the pre 2001 regime, we find that poor biosecurity was associated with the probability of reporting an outbreak. In the post 2001 regime, the risks changed, with trade being a much greater source of risk. We discuss the trade-off between trade restrictions and biosecurity measures in the management of FMD disease risks.

**Electronic supplementary material:**

The online version of this article (10.1007/s10393-018-1315-8) contains supplementary material, which is available to authorized users.

## Introduction

A growing number of zoonotic and epizootic diseases have been spread through international trade in livestock and wildlife products (Karesh et al. 2005; Fevre et al. 2006; Drew 2011; Karesh et al. 2012). Indeed, the increased spread of zoonotic and epizootic diseases is argued to be among the most important side effects of the growth in world trade (Perrings et al. 2010; Perrings 2014). What commodities and which markets are most implicated in trade-related disease risks is, however, not always intuitive. While imports of live animals from disease-endemic countries are an obvious source of risk, there is evidence (a) that exports to disease-endemic countries can be as great a source of risk as imports (Mur et al. 2012; Shanafelt and Perrings 2017), (b) that risks are highly sensitive to the volume of trade (Pavlin et al. 2009), and (c) that biosecurity measures in the export sector may offset the risks posed by disease endemicity (Heikkilä 2011).

**Fig. 1:**
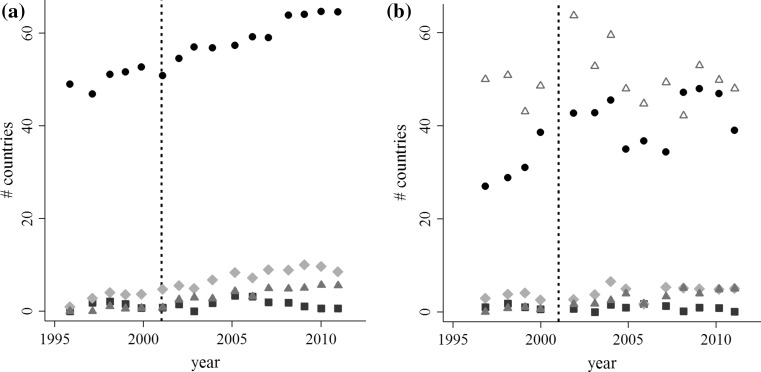
Global trends in the number of countries with OIE disease-free designations (**a**) and the number countries of each designation in the data (**b**). Symbol and color indicate the disease-free category: disease-free everywhere, no vaccination (black, circle); disease-free everywhere, vaccination (square, blue); disease-free zones, no vaccination (diamond, green); disease-free zones, vaccination (triangle, orange); not disease-free (hollow triangle, red). The dashed line marks the 2001 UK epidemic

**Fig. 2:**
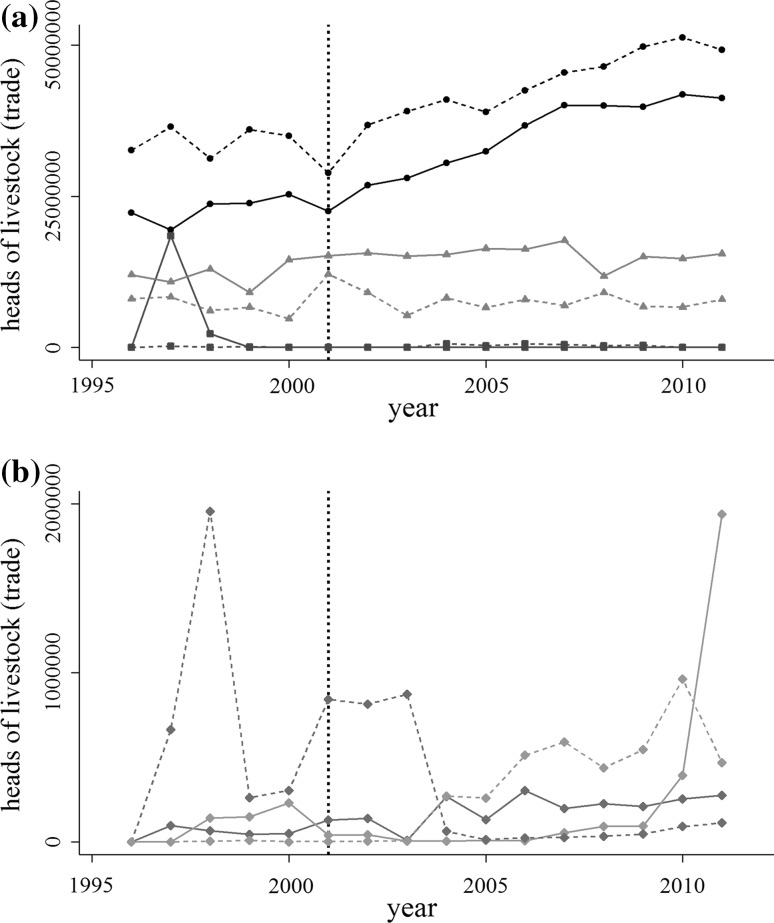
Imports (solid) and exports (dashed) of all heads of livestock aggregated by disease-free category. Color indicates the disease-free category. **a** Disease-free everywhere, no vaccination (black); disease-free everywhere, vaccination (blue); not disease-free (red). **b** Disease-free zones, no vaccination (green); disease-free zones, vaccination (orange). The dashed line indicates the 2001 UK epidemic

In this paper, we focus on the foot and mouth disease (FMD) risks of the international trade in livestock, focusing on the way in which disease risks have changed since the shift in trade-related disease risk management introduced after the 2001 UK outbreak. The 2001 UK outbreak marked a global shift in FMD management from trade bans toward trade access supported by enhanced biosecurity (Figs. 1, 2). Since 2001, many FMD-endemic countries have sought to expand their markets through the development of disease-free production zones and ancillary biosecurity measures (OIE and FAO 2012). The number of countries recognized by the World Organization of Animal Health (OIE) to be sufficiently “disease-free” to engage in the international trade in live animals has risen steadily since 2001 (Fig. 1a), as has the volume of goods traded by those countries (Fig. 2).

Trade interdictions remain an integral part of the international disease risk management regime. The dominant response to an outbreak is to manage local spread via culling infected and potentially infected animals and to ban imports of high-risk products (Grubman et al. 2008). The international management of trade-related disease risks is regulated by the Sanitary and Phytosanitary (SPS) Agreement, the standards for which (for FMD) are set by the OIE. Trade interventions are required to undergo a scientific risk assessment to establish the likelihood of the introduction, establishment, and spread of disease within an importing country, and to assess the biological and economic risks of infection (MacDiarmid 2011). For many FMD-endemic countries, exports are permanently banned. For others, the OIE applies the principles of the Terrestrial Animal Health Code (TAHC), which allows exports from disease-free production zones that have been geographically isolated from infected or endemic zones (OIE 2015).

There are criticisms that the geographically based regime negatively impacts environmental and social well-being in developing countries (Thomson et al. 2013a, b). The effectiveness of the post 2001 regime in managing disease risks has not, however, been questioned. While there are a number of efforts to quantify the risks of FMD outbreaks before and after 2001 (Berentsen et al. 1992; Garner and Lack 1995; Hartnett et al. 2007; Martinez-Lopez et al. 2008; Jori et al. 2009; Miller et al. 2012), we are not aware of any study that investigates the effect of the change in global FMD management that followed the 2001 UK epidemic.

We parameterize a model of disease risk as a function of trade structure and biosecurity, controlling for the presence of endemic wild reservoirs. Specifically, we estimate the relation between trade volumes, biosecurity measures, and the existence of wild reservoirs on the probability of disease outbreaks. In the post 2001 regime, application of a compartmental approach to disease management has allowed a significant increase in trade volumes from countries that were previously excluded from the market because of the existence of wild reservoirs. We are particularly interested in the disease risks of trade from countries declared to be disease-free (vaccination or no vaccination strategies), and in seeing how these risks changed after 2001.

## Data Description

Our dataset spans 138 countries between the period of 1997 and 2011. It includes the number of reported monthly outbreaks published by the World Organization of Animal Health’s Handistatus II and WAHID databases (http://www.oie.int/). The OIE includes 180 member countries, but reports outbreaks in both member and non-member countries. We cannot exclude the possibility that there is under-reporting of outbreaks, but we note that low-risk and high-risk countries are both represented in our data (Fig. 1b). Using the OIE data, we constructed an annual binary new outbreak(s)/no outbreak measure for each country. This is the primary dependent variable in our analysis.

We expect the likelihood of a country experiencing an outbreak to be positively correlated with the volume of trade with high-risk countries (e.g., imports from and exports to high-risk countries), and the presence of an endemic disease reservoir within a country. We expect it to be negatively correlated with the economic consequences of outbreaks, and the biosecurity measures in place in importing countries.

We considered three proxies for the economic consequences of an outbreak, which we collectively refer to as “value at risk.” The first is agricultural GDP as reported by the Food and Agriculture Organization (FAO). This is a direct measure of the value of goods and services produced by agriculture sector. Since it not possible to isolate livestock, this is an overestimate of the stream of benefits from agricultural stocks. The second is the FAO’s livestock production index (LPI). We considered this to be a proxy for the development of a country’s livestock sector. It is calculated as a country’s aggregate volume of production compared to a base period (between 2004 and 2006). The third is a measure of the assets that may be destroyed during efforts to control an outbreak—the aggregate standing stock of cattle, sheep, and pigs.

Our trade dataset included the volume of imports and exports of all cloven-hoofed animals reported to the Food and Agriculture Organization between 1996 and 2011 (http://faostat3.fao.org/home/E). We grouped imports and exports of countries by their disease status at the OIE: disease-free (no vaccination), disease-free (vaccination), disease-free zones (no vaccination), disease-free zones (vaccination), and not disease-free. The volume of imports captures the direct impact of trade on disease risk, e.g., the probability of importing an infected animal. The volume of exports captures the indirect effect of trade on disease risk—the probability that sending livestock transport vessels into high-risk ports will lead to outbreaks in the exporting country. That is, there is some probability that infected materials are “picked up” and transported back to the exporting country (Mur et al. 2012). This is equivalent to the global spread of invasive species in ballast water or transport containers (Hulme 2009). Since the FMD virus has been found to persist in hay, soil, fodder, milk, hair, machinery, and clothing, this may be a significant source of risk (Callis 1996; Alexandersen et al. 2003; Sutmoller et al. 2003; Sutmoller and Casas Olascoaga 2003; Ryan et al. 2008; Paton et al. 2010).

**Fig. 3:**
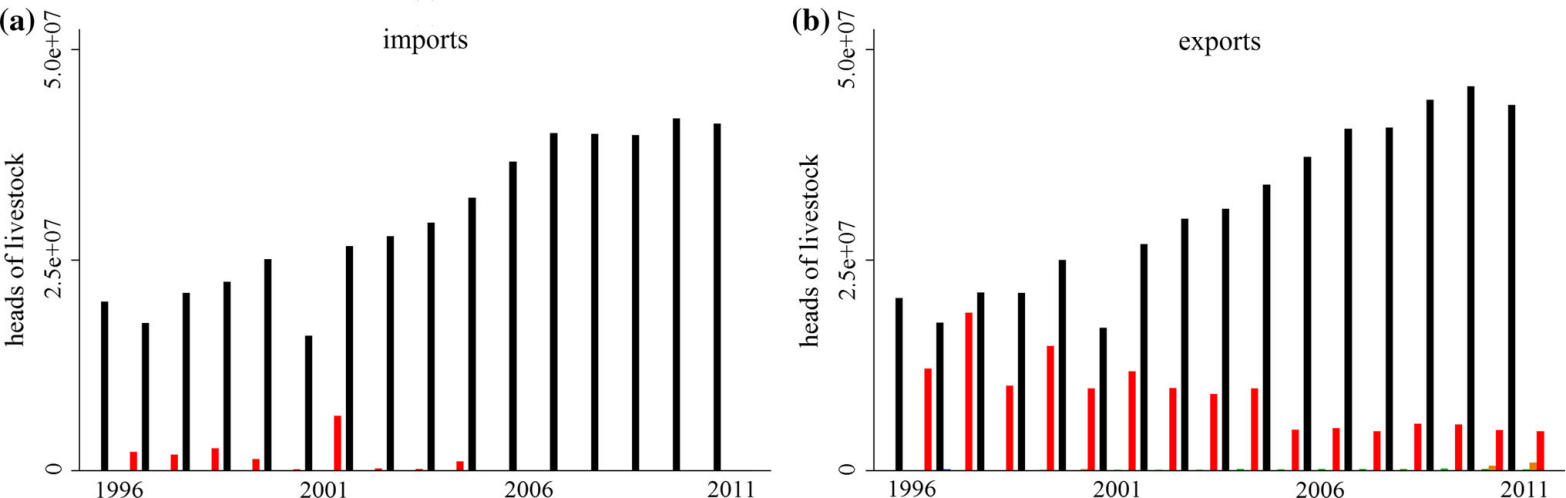
Aggregate imports from and exports to countries with a disease-free everywhere, no vaccination designation. Color indicates trade with countries of a particular disease-free status: disease-free, no vaccination (black), disease-free, vaccination (blue), disease-free zones, no vaccination (green), disease-free zones, vaccination (orange), and not disease-free (red)

**Fig. 4:**
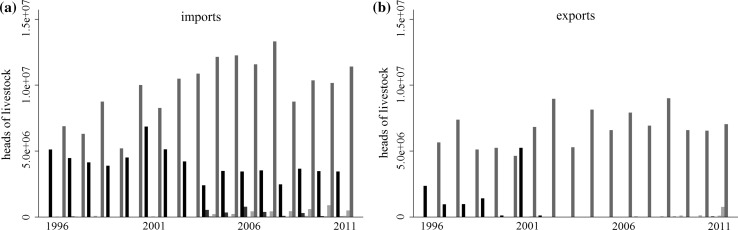
Aggregate imports from and exports to countries without a disease-free designation. Color indicates trade with countries of a particular disease-free status: disease-free, no vaccination (black), disease-free, vaccination (blue), disease-free zones, no vaccination (green), disease-free zones, vaccination (orange), and not disease-free (red)

**Fig. 5:**
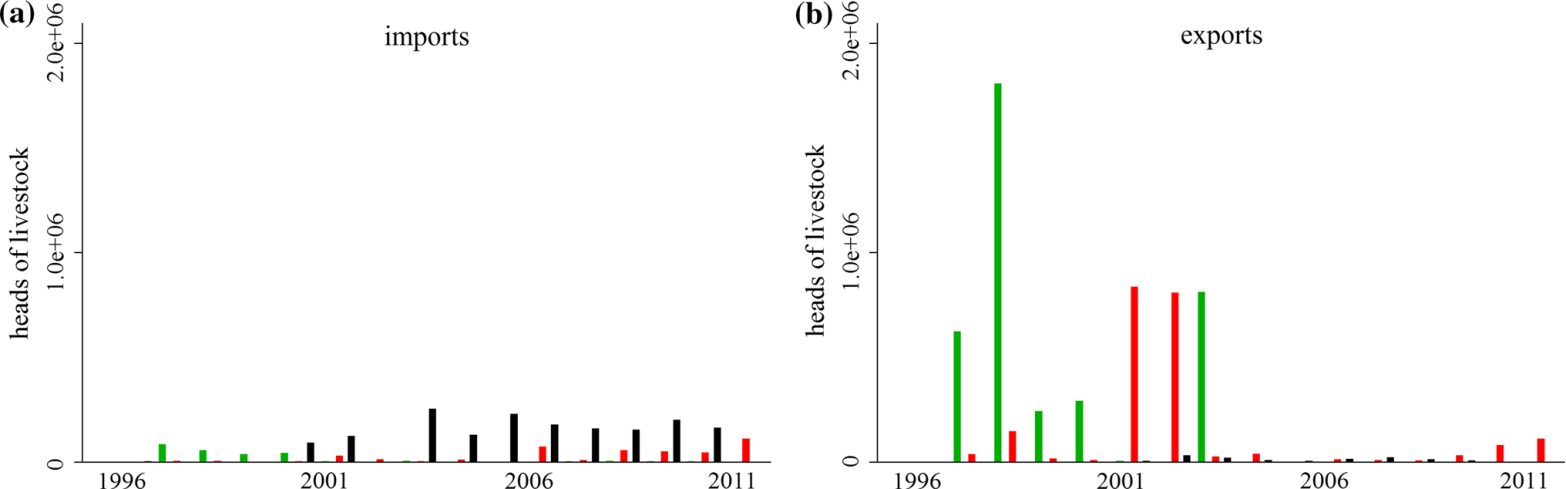
Aggregate imports from and exports to countries with disease-free zones, no vaccination designation. Color indicates trade with countries of a particular disease-free status: disease-free, no vaccination (black), disease-free, vaccination (blue), disease-free zones, no vaccination (green), disease-free zones, vaccination (orange), and not disease-free (red)

**Fig. 6:**
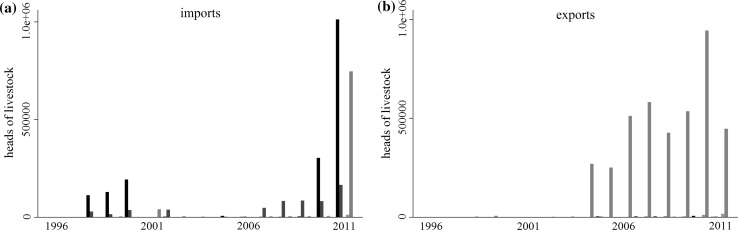
Aggregate imports from and exports to countries with disease-free zones, vaccination designation. Color indicates trade with countries of a particular disease-free status: disease-free, no vaccination (black), disease-free, vaccination (blue), disease-free zones, no vaccination (green), disease-free zones, vaccination (orange), and not disease-free (red)

Each disease-free designation reflects a particular trade and biosecurity regime. Countries that have a high disease-free designation (low risk of FMD) are likely to behave in ways that maintain that designation. We had expected that countries with high or low disease-free designations would trade only with each other, e.g., that low (high)-risk countries would trade only with other low (high)-risk countries. In reality, however, there is a large quantity of trade between countries with different disease-free designations (Figs. 3, 4, 5, and 6).

Our biosecurity dataset included proxies for the biosecurity elements thought to affect the likelihood of outbreaks (Berentsen et al. 1992; Garner and Lack 1995; Schoenbaum and Disney 2003). Unfortunately, direct measures of sanitary capacity are scarce. As a proxy, we included the density of veterinarians registered in each country with the OIE. For biosecurity measures, we included binary data on: (1) inspection and interception at the border; (2) monitoring and surveillance of livestock; (3) the existence of measures for the control of wild reservoirs; and (4) the presence of measures, such as veterinary cordon fences, that isolate disease-free regions within the country (http://www.oie.int). The last two are also proxies for the existence of wild reservoirs, which can affect commercial disease incidence rates if they are not isolated (Sutmoller et al. 2003; Thomson et al. 2003; Jori et al. 2009).

Finally, we included a second set of binary variables that reflect the disease management practices within a country and that contribute to its disease-free designation: the practice of vaccination and the prohibition of vaccination (http://www.oie.int) (OIE 2015). We expected countries that prohibit vaccination to be more cautious than countries that vaccinate the national herd. We also expected countries that meet the requirements for a high disease-free designation to seek to maintain that designation. Table 1 reports the summary statistics of the data included in our analysis. A list of all countries in the data may be found in the Electronic Supplementary Material.

**Table 1:** Summary of Outbreak Data and Independent Variables

Variable	Units	Proportion
FMD outbreak	binary	0.28

Variable	Units	Mean	SD	Min	Max
Agriculture value added	current US $billion	12.60	43.00	0.01	735.00
Livestock production index	–	100.81	14.95	55.10	182.83
Veterinarian density	#/km^2^	0.10	0.50	8.58E-7	5.47
Stocks (cattle, pigs, sheep)	# million heads	23.20	70.40	0.01	775.00

Variable	Units	Proportion
Existence of wild reservoirs	binary	0.07
Monitoring	binary	0.32
Precautions at the border	binary	0.67
Vaccinations practiced	binary	0.32
Vaccinations prohibited	binary	0.33
Zoning	binary	0.23

The livestock production index is a unit-less index of the aggregate volume of production of a country’s livestock sector compared to a baseline (in this case, the production between 2004 and 2006). Units are rounded to two decimal places

## Methods

There are a number of options available to test the effects of policy-induced structural breaks in data. These include the specification of interaction terms between explanatory variables and binary time indicators and the estimation of separate period models. Since the former involves substantive data loss required to standardize the data, we elected to split the data and estimate the same model for each of two periods: 1997–2000 and 2002–2011.

The options for modeling binary data in the literature include ordinary least squares regression (e.g., a linear probability model) (Cameron and Trivedi 2005), logit, probit, and tobit (Cameron and Trivedi 2005; Gelman and Hill 2007; Train 2009), and zero-inflated negative binomial (Zou 2004). Under specific sets of conditions, linear probability models can be useful explanatory tools for analysis (Caudill 1988; Horrace and Oaxaca 2006). However, in addition to being subject to the general assumptions of OLS, estimates can be less than zero or exceed one, model estimation can be numerically unstable, and efficiency gains can be small compared to other methods (Cameron and Trivedi 2005). Probit and tobit models can produce similar predicted probabilities as the logit, but use a different probability distribution function which can lead to differences when the predicted probabilities lie near the tails (Cameron and Trivedi 2005; Train 2009). Negative binomial regressions have been applied to zero-inflated binary data (Zou 2004), but the model is highly nonlinear and estimation difficult, particularly when using binary data (Allison and Waterman 2002). The logistic regression has the advantage in that it is standard across disciplines and can be extended to accommodate many complexities arising in statistical analysis including endogeneity of variables and autocorrelation (Cameron and Trivedi 2005; Gelman and Hill 2007; Train 2009). Therefore, we elected to implement a logit model of the form:

1 $$ \Pr (y_{it} = 1) = \left[ {1 + \exp \left( { - \theta_{it} } \right)} \right]^{ - 1} $$

2 $$ \theta_{it} = \alpha_{i} + \sum\limits_{j = 1}^{10} {Z_{jit}^{'} \beta_{j} } + \sum\limits_{k = 1}^{5} {M_{ikt}^{'} \beta_{k + 10} + } \sum\limits_{k = 1}^{5} {X_{ikt}^{'} } \beta_{k + 15} $$

for country *i* in year *t*. The left-hand side of Eq. (1) is the probability that a new FMD outbreak was reported to the OIE conditional on the linear predictors: $y_{it} = 1$ when a country has experienced a new outbreak; $y_{it} = 0$ when no outbreaks were reported to the OIE. The elements of *Z* include the following: value at risk, the log of veterinarian density, and binaries for disease control measures and management factors contributing to a country’s disease-free designation. Elements of *M* and *X* include the aggregate imports and exports between the country *i* and disease-free “region” *k*. It is the aggregate number of livestock imported from (exported to) countries with disease-free designation *k* into (by) country *i*. In order to increase the magnitude of the effects of trade, volumes of all species were summed together. The constant intercept term is represented by *α*.

Our data form an unbalanced panel. The models were estimated using the method of maximum likelihood with heteroskedastic-robust standard errors in Stata 14.0 (StataCorp 2015). In order to correct for potential unobserved, spatially correlated effects in reporting and disease incidence, we explicitly include country as a random effect.

We also performed tests to check for model misspecification and serial autocorrelation. We conducted a link test to test for model misspecification. While the test identified the potential for misspecification, the magnitude of the effect was negligible and model fit (explanatory power) was significantly greater in the original specification. Tests for serial autocorrelation often assume linearity in the model (Drukker 2003). However, as a first approximation, we conducted a pairwise correlation between each explanatory variable and its one-year time-lagged counterpart. Although the correlations for border precautions, prohibition of vaccination, and zoning were statistically significant at the ten percent level, correlation coefficients never exceeded 0.15.

## Results

Our results for the periods from 1997 to 2000 and 2002 to 2011 are summarized in Table 2 and Tables S3 and S4 in the Electronic Supplemental Material. We found a marked difference in the statistically significant disease risk factors before and after 2001. Prior to 2001, biosecurity measures were most strongly related to the probability that a country would report an outbreak. Only imports from *disease*-*free, no vaccination* countries were statistically significant at the ten percent level. Veterinarian density and the prohibition of vaccination were negatively related to the probability of reporting outbreaks (*P* < 0.01), while monitoring and the practice of vaccination were both positively related to the probability of outbreaks (*P* < 0.10). In this period, the volume of trade in live animals was not a statistically significant source of risk, nor were our measures of value at risk, border precautions, zoning, or the existence of wild reservoirs.

**Table 2:** Logistic Regression Estimates of Exogenous Variables

Variable	Pre 2001		Post 2001	
	Odds ratio	*P* value	Odds ratio	*P* value
Agriculture value added	1.000	0.221	1.000	0.256
Livestock production index	0.976	0.208	0.998	0.884
Veterinarian density (log-transf.)	0.654	0.005	0.744	0.018
Existence of wild reservoirs	0.288	0.229	4.513	0.105
Monitoring	3.620	0.061	1.462	0.553
Precautions at the border	1.130	0.836	0.709	0.571
Vaccinations practiced	3.499	0.03	2.262	0.15
Vaccinations prohibited	0.021	<0.001	0.312	0.144
Zoning	1.487	0.71	5.891	0.028
Stocks (cattle, sheep, pigs)	1.000	0.366	1.000	0.83
Importing from				
Disease-free, no vaccination	1.002	0.458	0.998	0.008
Disease-free, vaccination	0.994	0.083	0.996	0.884
Disease-free zones, no vaccination	1.024	0.607	1.057	0.348
Disease-free zones, vaccination	3.495	0.235	1.018	0.164
Not disease-free	1.001	0.893	1.008	<0.001
Exporting to				
Disease-free, no vaccination	0.838	0.322	0.969	0.039
Disease-free, vaccination	0.268	0.488	3.757	0.331
Disease-free zones, no vaccination	0.991	0.124	0.995	0.001
Disease-free zones, vaccination	1.121	0.175	1.014	<0.001
Not disease-free	1.003	0.331	0.999	0.613
Constant	0.169	0.333	0.011	0.01
Number of observations	341		1016	
Number of countries	127		136	

Estimates are rounded to three decimal places. Veterinarian density (log-transformed) is interpreted as the change in the odds for a percent change in veterinarian density. Trade variables are interpreted as the change in the odds ratio for a 1000-unit increase (heads of livestock) in trade. Note that our estimates are reported as odds ratios as opposed to relative risk. Odds ratios provide less accurate approximations of relative risk when they are very close to zero or much greater than one (Davies et al. 1998; Zhang and Yu 1998; Cohen 2000). Standard errors and 95% confidence errors for our estimates may be found in Tables S3 and S4 of the Electronic Supplemental Material

In the period after 2001, the statistically significant risk factors changed. Of our biosecurity measures, veterinarian density and zoning were statistically significant at the ten percent level. The existence of wild reservoirs and practice and prohibition of vaccination were significant at the fifteen percent level. Instead we found a statistically significant relation between trade with countries of particular categories and disease risk. Imports of live animals from *disease*-*free*, *no vaccination* countries were negatively related to the probability of disease outbreaks (*P* < 0.01). Imports from countries with *no disease*-*free* designation were positively related to the probability of outbreaks (*P* < 0.01). These capture the direct effects of trade-mediated disease risk and imply that imports of risk materials from “safe” regions were risk decreasing, while imports from “not disease-free” regions were risk increasing. Similarly, exports of live animals to countries with *disease*-*free, no vaccination* or *disease*-*free zones, no vaccination* statuses were negatively correlated with disease risk (*P* < 0.05 and *P* < 0.01, respectively). However, exports of live animals to countries operating *disease*-*free zones with vaccination* were positively related to the probability of disease outbreaks (*P* < 0.01).

We had expected value at risk and border precautions to be significant risk factors. None of our measures of value at risk—agricultural GDP, the livestock production index, or stocks of cattle, sheep and pigs—were significant in either model. Nor were border precautions.

## Discussion

The post 2001 regime has the goal of increasing the participation of disease-endemic countries in global markets through the development of production areas that are isolated from wild or domestic FMD reservoirs, or protected through vaccination. In one respect, the policy has been successful. The number of countries with disease-free designations has increased (Fig. 1), and aggregate trade volumes increased by about 33% between 2001 and 2011, compared to a 2% decline between 1996 and 2001 (Fig. 2).

However, the shift in global management policy has also affected the global disease risk factors for FMD. Prior to 2001, we found that trade volumes were not statistically significant risk factors for FMD outbreaks and that the best predictors of the probability of outbreaks were veterinary capacity, disease monitoring, and vaccination policy. Countries with lax biosecurity and an endemic wild disease reservoir were more likely to report outbreaks.

After the 2001 UK outbreak, more effort was made to mitigate trade risks as non-disease-free countries sought to increase exports from biosecure production zones (OIE and FAO 2012). The most significant risk factors were no longer biosecurity measures, but trade. Of the biosecurity measures tested, only zoning was a statistically significant source of risk.

For trade, our results for countries at the extreme ends of the disease-free status spectrum are intuitive. Imports from/exports to low (high)-risk countries were negatively (positively) correlated with the probability of reporting an outbreak. For countries at intermediate disease-free designations, the risks of trade turned out to be less intuitive. We found that imports from countries with a *disease*-*free, vaccination* designation or from those with *disease*-*free zones* (with or without vaccination) were not statistically significant sources of risk of FMD. However, exports to countries operating *disease*-*free zones* were negatively or positively associated with the probability of reporting an outbreak, depending on the vaccination policy. The presence of disease-free zones implies the existence of an endemic disease reservoir in wild or commercial livestock populations, but biosecurity regimes based on vaccination imply a weaker commitment to the isolation of disease-free areas than no vaccination biosecurity regimes.

While we believe that we have controlled for the primary sources of bias, we cannot rule out the possibility of reporting bias, unobserved heterogeneity in the sample, and omitted variable bias. We observe representation from high- and low-risk countries in the data (Fig. 1), and local involvement of the OIE in member and non-member countries should lessen potential reporting bias. Unobserved, country-specific effects should be partially accounted for in the country-level random effect. Finally, we cannot exclude the possibility that there are other relevant factors for FMD spread. Live animals are not the only source of risk (Alexandersen et al. 2003; Arzt et al. 2011a, b), and we lack detailed data on shipping routes and trade volumes in other risk materials. It would be useful to consider trade in other risk materials that can potentially spread FMD, such as meat and milk.

There are concerns that the geographically focused biosecurity policies reflected in the Terrestrial Animal Health Code have negative effects on environmental and social well-being in developing countries (Thomson et al. 2013a, b). These focus primarily on countries in Africa (Thomson et al. 2013a), but they are by no means limited to them. In our data, the majority of the countries within the *disease*-*free zones* designations are developing countries in South America. While one of the long-term goals of the Global FMD Control Policy is to transition countries without a disease-free designation to those with disease-free zones (OIE and FAO 2012), the costs of developing and maintaining the required infrastructure to isolate disease-free zones can be quite high (Thomson et al. 2013b). This can create a positive feedback loop preventing developing countries from obtaining or keeping a disease-free designation. If an outbreak occurs, the country loses access to higher-value global markets making it more difficult to restore biosecurity and sanitary conditions required to re-establish trade. Indeed, the number of countries with a *disease*-*free zone* designation has remained more or less constant since 2005 (Fig. 1). Moreover, while countries with *disease*-*free, vaccination* status import from low-risk countries, most of their exports are directed to countries that are *not disease*-*free* (Fig. 6). Countries with *disease*-*free zones and vaccination* do not have access to the lucrative markets in countries of higher disease-free designations. At the same time, by trading with high-risk countries they are potentially exposing themselves and their trading partners to more risk (Shanafelt and Perrings 2017). We found that trade with countries combining disease-free zones and vaccination increased the probability of an FMD outbreaks.

Commodity-based trade standards have been proposed as an alternative to the geographically based standards of the OIE and Codex Alimentarius (Thomson et al. 2004, b). Commodity-based approaches focus on biosecurity and monitoring at all levels of the food chain—not just the point of origin of the initial product. The OIE and Codex Alimentarius have begun working on “whole food chain” approaches to food safety (Oidtmann et al. 2013), and the Food and Agriculture Organization has argued for the practicality of such an approach to managing disease risk (FAO 2011). However, the process needs to be refined to increase efficiency and lower costs (Thomson et al. 2013b), and its impact on disease risk needs to be quantified. While our results do not bear directly on the risk implications of this approach, we can say that trade into and out of countries where the disease is endemic is currently risk increasing. If a shift to a commodity-based approach were to be risk-neutral, it would likely require additional, albeit more targeted, biosecurity (Figs. 5, 6).

To conclude, there is a trade-off to be made between import and export restrictions and investments in biosecurity measures for the management of the disease risks of trade. The prohibition of risky imports or exports may be risk reducing, but it also imposes welfare costs on the countries affected. While the disease risks associated with the imports or export of risky commodities may be reduced through biosecurity measures, these too have costs. Managing trade-related disease risks requires a balance between the two. The management of trade-related FMD risks after the 2001 UK epidemic has addressed the balance by increasing the scope for developing countries to export but at the cost of increased biosecurity measures. Except in the case of exports from countries operating under a *disease*-*free zones, vaccination* regime, our results support the claim that this has been largely successful. Exports have grown while disease risks, or at least the odds ratios, have not. That said, disease-endemic countries using vaccination are a more significant source of disease risk in the post 2001 regime, and trade volumes are a better predictor of disease risk than biosecurity measures. Finally, the call for commodity-based approaches to biosecurity stems from the perception that the regime is still too restrictive. A commodity-based approach promises freer trade, but linked to commodity-specific biosecurity measures. Since commodity- and zone-based biosecurity measures have different costs and different impacts on disease risk, it is reasonable to ask which regime is most likely to lead to the highest net welfare gains.

## Electronic supplementary material

Below is the link to the electronic supplementary material.

Supplementary material 1 (DOCX 209 kb)
