# Supplementary material for: The Effect of the Post 2001 Reforms on FMD Risks of the International Live Animal Trade
Source: Ecohealth. 2018 Feb 27;15(2):327–37. doi: 10.1007/s10393-018-1315-8 (PMC6132411; doi:10.1007/s10393-018-1315-8)
Supplement: Supplementary file 1 — Supplementary material 1 (DOCX 209 kb) [file 10393_2018_1315_MOESM1_ESM.docx]

ELECTRONIC SUPPLEMENTARY MATERIAL

Table S1. List of Pre 2001 Countries in the Data

| Pre 2001 Countries |  |  |  |
| --- | --- | --- | --- |
|  |  |  |  |
| Albania | Denmark | Malaysia | Turkey |
| Algeria | Dominica | Mali | Turkmenistan |
| Angola | Dominican Republic | Malta | Uganda |
| Antigua and Barbuda | Ecuador | Mexico | United Kingdom |
| Argentina | Egypt | Nepal | United States of America |
| Armenia | El Salvador | Netherlands |  |
| Australia | Estonia | New Zealand | Uruguay |
| Austria | Ethiopia | Nicaragua | Uzbekistan |
| Azerbaijan | Fiji | Niger | Venezuela |
| Bahamas | Finland | Nigeria | St. Vincent and the Grenadines |
| Barbados | France | Norway |  |
| Belarus | French Polynesia | Papua New Guinea | Zambia |
| Belgium | Gabon | Paraguay | Zimbabwe |
| Belize | Georgia | Peru |  |
| Benin | Germany | Philippines |  |
| Bhutan | Ghana | Poland |  |
| Bolivia | Grenada | Portugal |  |
| Bosnia and Herzegovina | Guinea | Puerto Rico |  |
|  | Guyana | Romania |  |
| Botswana | Honduras | Russia |  |
| Brazil | Hungary | Rwanda |  |
| Brunei Darussalam | Iceland | Slovakia |  |
| Bulgaria | India | Slovenia |  |
| Burkina Faso | Indonesia | South Africa |  |
| Burundi | Ireland | Spain |  |
| Cameroon | Italy | Sri Lanka |  |
| Chad | Jamaica | St. Kitts and Nevis |  |
| Chile | Japan | Suriname |  |
| China | Kazakhstan | Swaziland |  |
| Colombia | Kenya | Sweden |  |
| Costa Rica | Kyrgyzstan | Switzerland |  |
| Cote d'Ivoire | Latvia | Tajikistan |  |
| Croatia | Lebanon | Tanzania |  |
| Cuba | Lesotho | Thailand |  |
| Cyprus | Lithuania | Togo |  |
| Czech Republic | Luxembourg | Trinidad and Tobago |  |
| Democratic Republic of the Congo | Madagascar | Tunisia |  |
|  | Malawi | Ukraine |  |
|  |  |  |  |

Table S2. List of Post 2001 Countries in the Data

| Post 2001 Countries |  |  |  |
| --- | --- | --- | --- |
|  |  |  |  |
| Albania | Czech Republic | Madagascar | Spain |
| Algeria | Democratic Republic of the Congo | Malawi | Sri Lanka |
| Angola |  | Malaysia | St. Kitts and Nevis |
| Antigua and Barbuda | Denmark | Mali | St. Vincent and the Grenadines |
| Argentina | Dominica | Malta |  |
| Armenia | Dominican Republic | Mauritius | Suriname |
| Australia | Ecuador | Mexico | Swaziland |
| Austria | Egypt | Mongolia | Sweden |
| Azerbaijan | El Salvador | Morocco | Switzerland |
| Bahamas | Estonia | Lithuania | Syria |
| Barbados | Ethiopia | Luxembourg | Tajikistan |
| Belarus | Fiji | Montenegro | Tanzania |
| Belgium | Finland | Mozambique | Suriname |
| Belize | France | Namibia | Swaziland |
| Benin | Gabon | Nepal | Sweden |
| Bhutan | Georgia | Netherlands | Switzerland |
| Bolivia | Germany | New Zealand | Syria |
| Bosnia and Herzegovina | Ghana | Nicaragua | Tajikistan |
|  | Greece | Niger | Tanzania |
| Botswana | Grenada | Nigeria | Thailand |
| Brazil | Guatemala | Norway | Togo |
| Brunei Darussalam | Guinea | Papua New Guinea | Trinidad and Tobago |
| Bulgaria | Guyana | Paraguay | Tunisia |
| Burkina Faso | Honduras | Peru | Turkey |
| Burundi | Hungary | Philippines | Turkmenistan |
| Cameroon | Iceland | Poland | Uganda |
| Canada | India | Portugal | Ukraine |
| Central African Republic | Indonesia | Romania | United Kingdom |
|  | Ireland | Russia | United States of America |
| Chad | Italy | Rwanda |  |
| Chile | Jamaica | Sao Tome and Principe | Uruguay |
| China | Japan |  | Uzbekistan |
| Colombia | Kazakhstan | Senegal | Venezuela |
| Costa Rica | Kenya | Serbia | Zambia |
| Cote d'Ivoire | Kyrgyzstan | Sierra Leone | Zimbabwe |
| Croatia | Latvia | Slovakia |  |
| Cuba | Lebanon | Slovenia |  |
| Cyprus | Lesotho | South Africa |  |
|  |  |  |  |

Table S3. Full Pre 2001 Logistic Regression Estimates of Exogenous Variables

|  |  |  |  |  |  |  |  |  |  |
| --- | --- | --- | --- | --- | --- | --- | --- | --- | --- |
|  | Variable |  | Odds ratio | *P* value |  | SE | 95% confidence interval | |  |
|  | Agriculture value added |  | 1.000 | 0.221 |  | <0.001 | 1.000 | 1.000 |  |
|  | Livestock production index |  | 0.976 | 0.208 |  | 0.019 | 0.939 | 1.014 |  |
|  | Veterinarian density |  | 0.654 | 0.005 |  | 0.010 | 0.485 | 0.882 |  |
|  | Existence of wild reservoirs |  | 0.288 | 0.229 |  | 0.298 | 0.038 | 2.187 |  |
|  | Monitoring |  | 3.620 | 0.061 |  | 2.485 | 0.943 | 13.903 |  |
|  | Precautions at the border |  | 1.130 | 0.836 |  | 0.668 | 0.355 | 3.599 |  |
|  | Vaccinations practiced |  | 3.499 | 0.03 |  | 2.017 | 1.130 | 10.832 |  |
|  | Vaccinations prohibited |  | 0.021 | <0.001 |  | 0.018 | 0.004 | 0.117 |  |
|  | Zoning |  | 1.487 | 0.71 |  | 1.587 | 0.184 | 12.048 |  |
|  | Stocks (cattle, sheep, pigs) |  | 1.000 | 0.366 |  | <0.001 | 1.000 | 1.000 |  |
|  |  |  |  |  |  |  |  |  |  |
| Importing from: | Disease-free, no vaccination |  | 1.002 | 0.458 |  | 0.003 | 0.996 | 1.008 |  |
|  | Disease-free, vaccination |  | 0.994 | 0.083 |  | 0.003 | 0.988 | 1.001 |  |
|  | Disease-free zones, no vaccination |  | 1.024 | 0.607 |  | 0.047 | 0.935 | 1.122 |  |
|  | Disease-free zones, vaccination |  | 3.495 | 0.235 |  | 3.680 | 0.444 | 27.517 |  |
|  | Not disease-free |  | 1.001 | 0.893 |  | 0.009 | 0.984 | 1.019 |  |
|  |  |  |  |  |  |  |  |  |  |
| Exporting to: | Disease-free, no vaccination |  | 0.838 | 0.322 |  | 0.149 | 0.591 | 1.189 |  |
|  | Disease-free, vaccination |  | 0.268 | 0.488 |  | 0.509 | 0.006 | 11.105 |  |
|  | Disease-free zones, no vaccination |  | 0.991 | 0.124 |  | 0.006 | 0.979 | 1.003 |  |
|  | Disease-free zones, vaccination |  | 1.121 | 0.175 |  | 0.094 | 0.951 | 1.321 |  |
|  | Not disease-free |  | 1.003 | 0.331 |  | 0.003 | 0.997 | 1.010 |  |
|  |  |  |  |  |  |  |  |  |  |
|  | Constant |  | 0.169 | 0.333 |  | 0.311 | 0.005 | 6.185 |  |
|  |  |  |  |  |  |  |  |  |  |

Estimates are rounded to three decimal places. Standard errors (SE) are calculated as Huber/White sandwich errors to correct for heteroskedasticity

Table S4. Full Post 2001 Logistic Regression Estimates of Exogenous Variables

|  |  |  |  |  |  |  |  |  |  |
| --- | --- | --- | --- | --- | --- | --- | --- | --- | --- |
|  | Variable |  | Odds ratio | *P* value |  | SE | 95% confidence interval | |  |
|  | Agriculture value added |  | 1.000 | 0.256 |  | <0.001 | 1.000 | 1.000 |  |
|  | Livestock production index |  | 0.998 | 0.884 |  | 0.015 | 0.970 | 1.027 |  |
|  | Veterinarian density |  | 0.744 | 0.018 |  | 0.093 | 0.582 | 0.950 |  |
|  | Existence of wild reservoirs |  | 4.513 | 0.105 |  | 4.189 | 0.732 | 27.839 |  |
|  | Monitoring |  | 1.462 | 0.553 |  | 0.936 | 0.417 | 5.125 |  |
|  | Precautions at the border |  | 0.709 | 0.571 |  | 0.431 | 0.215 | 2.331 |  |
|  | Vaccinations practiced |  | 2.262 | 0.15 |  | 1.282 | 0.745 | 6.869 |  |
|  | Vaccinations prohibited |  | 0.312 | 0.144 |  | 0.249 | 0.066 | 1.487 |  |
|  | Zoning |  | 5.891 | 0.028 |  | 4.756 | 1.211 | 28.666 |  |
|  | Stocks (cattle, sheep, pigs) |  | 1.000 | 0.830 |  | <0.001 | 1.000 | 1.000 |  |
|  |  |  |  |  |  |  |  |  |  |
| Importing from: | Disease-free, no vaccination |  | 0.998 | 0.008 |  | 0.001 | 0.997 | 0.999 |  |
|  | Disease-free, vaccination |  | 0.996 | 0.884 |  | 0.025 | 0.948 | 1.047 |  |
|  | Disease-free zones, no vaccination |  | 1.057 | 0.348 |  | 0.063 | 0.941 | 1.188 |  |
|  | Disease-free zones, vaccination |  | 1.018 | 0.164 |  | 0.013 | 0.993 | 1.043 |  |
|  | Not disease-free |  | 1.008 | <0.001 |  | 0.002 | 1.005 | 1.011 |  |
|  |  |  |  |  |  |  |  |  |  |
| Exporting to: | Disease-free, no vaccination |  | 0.969 | 0.039 |  | 0.015 | 0.941 | 0.998 |  |
|  | Disease-free, vaccination |  | 3.757 | 0.331 |  | 5.113 | 0.261 | 54.108 |  |
|  | Disease-free zones, no vaccination |  | 0.995 | 0.001 |  | 0.002 | 0.991 | 0.998 |  |
|  | Disease-free zones, vaccination |  | 1.014 | <0.001 |  | 0.003 | 1.009 | 1.019 |  |
|  | Not disease-free |  | 0.999 | 0.613 |  | 0.001 | 0.997 | 1.002 |  |
|  |  |  |  |  |  |  |  |  |  |
|  | Constant |  | 0.011 | 0.01 |  | 0.019 | <0.001 | 0.335 |  |
|  |  |  |  |  |  |  |  |  |  |

Estimates are rounded to three decimal places. Standard errors (SE) are calculated as Huber/White sandwich errors to correct for heteroskedasticity
